# Air pollution as cause of mental disease: Appraisal of the evidence

**DOI:** 10.1371/journal.pbio.3000370

**Published:** 2019-08-20

**Authors:** John P. A. Ioannidis

**Affiliations:** Meta-Research Innovation Center at Stanford (METRICS) and Stanford Prevention Research Center, Departments of Medicine, Health Research and Policy, Biomedical Data Science, and Statistics, Stanford University, Stanford, California, United States of America

## Abstract

A causal association of air pollution with mental diseases is an intriguing possibility raised in a Short Report just published in *PLOS Biology*. Despite analyses involving large data sets, the available evidence has substantial shortcomings, and a long series of potential biases may invalidate the observed associations. Only bipolar disorder shows consistent results, with similar effects across United States and Denmark data sets, but the effect has modest magnitude, appropriate temporality is not fully secured, and biological gradient, plausibility, coherence, and analogy offer weak support. The signal seems to persist in some robustness analyses, but more analyses by multiple investigators, including contrarians, are necessary. Broader public sharing of data sets would also enhance transparency.

## Abstract 2

The association of air pollution with mental health is an intriguing possibility suggested by an analysis of two large data sets from the US and Denmark. This Primer provides context and caveats to this study in order to guide readers as to how to interpret it.

The search for causes of mental diseases is notoriously difficult. Mental disorders are challenging to define and measure. Nongenetic factors may be a major determinant for their occurrence [1–3]. However, the implicated exposures are heavily correlated and/or confounded. Moreover, time lag, dose–response, and susceptible periods in life can only be speculated about. When addressing the potential causal effects of air pollution on mental health, these problems become all too obvious. Therefore, the study by Khan and colleagues [4] is a valuable contribution. Using large-scale data from the United States and Denmark, the authors demonstrate correlation signals between air pollution and bipolar disease in both countries and between air pollution and depression, schizophrenia, and personality disorder in Denmark. The US data also show correlation between poor land quality and personality disorder (an association not assessed in Denmark).

## Data and design issues

Are the data that generate these signals and the study design employed suitable to answer this type of research question? Both data sets harness enormous sample sizes, but this offers no guarantee of validity. Analysis of big data can draw absurd conclusions because of fundamental deficiencies in the quality of the data [5]. The US database is approximately 100-fold larger than the Danish one, but the latter is of better quality.

The US IBM MarketScan data set encompasses a population segment that is already heavily selected for by being insured and having medical claims. The measurement of air pollution exposures is at the county level, generating an ecological design subject to ecological fallacies [6]. Mental disease diagnoses depend on their accurate or inaccurate recording in medical claims. There is no validation of these data against some gold standard; e.g., such validation would entail the comparison (at least in a sample of people) of the ecological exposures against carefully collected individual exposures and the comparison of claims-based disease codes against carefully adjudicated diagnoses. A crude reality check is offered by visualizing the data and by comparing the prevalence of each disease against what is generally known. Data visualizations do not suggest any major artifacts but are able to pick up only dramatic errors and biases. As for disease prevalence, the numbers look quite appropriate, except for personality disorders, for which the reported prevalence (0.15%) seems less than one-tenth of what are reasonable population prevalence values. Perhaps not surprisingly, most personality disorders remain unreported in medical claims data. Given these caveats, results from the US data offer mostly coarse, exploratory hints. Associations may be entirely spurious or, conversely, important associations may be missed because of these deficiencies.

The Danish data seem more robust. The entire country population is sampled, missingness is limited, and exposures are measured for individuals. Accuracy of mental disease phenotypes is still less than optimal, given the difficulty of establishing these diagnoses, but not worse than in the US data.

In some other dimensions, the two data sets differ in ways that may or may not make one better than the other. In the US data, the exposure has been measured in 2000–2005, while the mental disease diagnoses are captured in 2003–2013. Conversely, the Danish data assess air pollution in the first 10 years of life with mental disease diagnosed up to age 14–37 (depending on when exactly each person was born). Thus, the Danish data assess whether childhood exposure increases the risk of early diagnosed mental disease, while the US data assess whether air pollution increases mental disease risk within a few years, mostly in adults. These are different scientific questions, and answers may genuinely diverge. Second, exposure measurements are more complex in the US data, with 87 variables included in calculating a first principle component for air pollution, while only 14 variables are considered in Denmark (and only 6 variables are included in both data sets). It is uncertain whether it is better to have more or fewer variables to define air pollution, let alone if only some (and if so, which) of these correlated variables might cause disease.

## Bradford Hill criteria

After acknowledging these background considerations, one can now examine how the evidence of Khan and colleagues fares regarding the classic Bradford Hill criteria for causation [7] (Table 1).

**Table 1:** Bradford Hill criteria/considerations for the association between air pollution and mental health and, for comparison, between air pollution and mortality.

*Criteria/Consideration*	*Mental Health*	*Mortality*
Strength	+/–	+/–
Consistency	– (+ for bipolar)	+
Specificity	–	–
Temporality	+/–	+
Biological gradient	+	+
Plausibility	+/–	+
Coherence	(+)	+
Experiment	–	–
Analogy	(+)	(+)

+, criterion mostly fulfilled; +/−, criterion fulfilled but caveats exist; −, criterion not fulfilled; (+), criterion likely to be at best weakly informative in this setting.

The strength of the observed associations is modest. Effect sizes are larger in the Danish data but never reach relative risks of >3. However, this is not necessarily a weakness. It has been argued [8] that most true causal effects are modest or even small/tiny; too-large effects may simply point to errors and biases. 30%–40% relative risk increases in the risk of bipolar disease or also other phenotypes are reasonable.

Consistency across the two data sets is debatable. Khan and colleagues seem to interpret their results as being replicated across countries. However, with the exception of bipolar disorder, the point estimates are strikingly dissimilar between the US and Denmark, and 95% confidence intervals do not even overlap.

Specificity is almost impossible to ask for in probing causes of mental disease. Patterns of causation are likely to be complex and nonspecific because these diseases are correlated and overlapping between each other, and the same applies for environmental exposures [9].

Temporality is only modestly secured, given the study design on both the US and Denmark analyses. Measurements of exposure to air pollution mostly precede the psychiatric diagnoses. However, exceptions may exist, e.g., IBM MarketScan diagnoses made in 2003–2005 may predate some air pollution exposure data. Moreover, recorded dates of psychiatric disease diagnosis may not represent disease onset. The disease process may have started years earlier.

Biological gradient is present in almost all the signals detected with gradual increase in risk across septiles of exposure. Nevertheless, the choice of septiles for the analyses is weird, and one wonders whether the analysts picked this option in advance: a search in PubMed (6/15/2019) with tertile*, quartile*, and quintile* yields 13,606, 28,267, and 11,283 items, respectively, while a search with septile* yields only 16. Emphasis on relative risks for the contrast of the two extreme septiles yields numerical figures that seem sizeable but that do not represent the experience of most people.

Plausibility is one of the most difficult criteria to operationalize. Khan and colleagues review the biological literature and mention several studies highlighting biological mechanisms for an association of air pollution with neuroinflammation and neurotoxicity in both bipolar disorder and depression. However, the problem with such in vivo and in vitro data is that almost always, they are not systematically collected. Their publication is usually driven by the urge to present convincing “narratives.” Selection biases in this literature are probably rampant but extremely difficult to quantify, given the lack of registration or widely accepted rules on what should be reported [10].

Coherence is also difficult to operationalize. Nevertheless, our knowledge about mental diseases and their biology and natural history does not conflict seriously with the observed signals.

None of the detected signals are based on experiment. It is certainly unethical to randomize individuals or communities to air pollution. These are observational data with all the limitations involved in analyzing them. Efforts have been made to address confounding; however, residual confounding must be extensive. In the IBM MarketScan data, the ecological design poses even further limitations. Even the key known potential confounders, e.g., race/ethnicity, are adjusted for only as county-level percentages.

Analogy is another debatable criterion. Air pollution has evidence for association with several other conditions, e.g., respiratory disease [11] and overall mortality [12]. Also, mental diseases may depend on multiple other environmental exposures and experiences [1–3]. However, this does not prove necessarily that air pollution should necessarily also affect mental diseases.

Table 1 summarizes each of the 9 criteria for the proposed association with mental diseases and also, for comparison, for the association between air pollution and overall mortality, for which evidence is much stronger.

## Rating of evidence based on quantitative criteria

A different, complementary set of criteria has been proposed for assessing the strength of observational evidence for putative risk factors when multiple studies are available and can be summarized in meta-analyses [13]. In the case of environmental risk factors and mental health, several systematic reviews and/or meta-analyses [14–16] and also umbrella reviews synthesizing multiple meta-analyses exist [1–3]. However, prior evidence on pollution is scant compared with the Khan and colleagues results. Prior overviews lack other good data on bipolar disorder and air pollution, and the limited data on schizophrenia are too heterogeneous to synthesize formally [16]. Table 2 juxtaposes the evidence for bipolar disorder and schizophrenia based on the synthesis of the two studies presented by Khan and colleagues. Both in the US and in Denmark, the *P*-values even for the contrast of the extreme septiles do not reach *P* < 10^−6^. Furthermore, for schizophrenia, the very large difference between the Denmark and US results does not allow yet high confidence for the validity of an association.

**Table 2:** Quantitative criteria for the strength of the evidence for air pollution and risk of bipolar disorder and schizophrenia.

*Criteria*	*Bipolar Disorder*	*Schizophrenia*
Large amount of evidence		
Data on >1,000 disease diagnoses	+	+
Strong statistical support		
*P*-value < 10^−3^ (random effects)	+	–
*P*-value < 10^−6^ (random effects)	–	–
No large heterogeneity		
Heterogeneity I^2^ < 50%	+	–
95% PI excludes the null	N/A	N/A
Largest study shows an effect		
*P* < 0.05 in the largest study	+	–
No obvious hints of selective reporting		
No small study effects detected	N/A	N/A
No excess significance detected	N/A	N/A

N/A: cannot calculate given the small number of studies; only the two estimates from US and Denmark from Khan and colleagues are considered here. PI calculation would require at least 3 studies with independent effect estimates to be available. Small study effects and excess significance tests would provide hints (not proof) for possible selection and other publication biases, but these tests require many studies with published estimates to be available in order to be assessed with any reliability. It is unknown how many other investigators may have tried to evaluate the association of air pollution and these mental health phenotypes. For reviews of previous studies of environmental exposures, see [14,16]. **Abbreviations:** N/A, not available; PI, prediction interval.

## Transparency, reproducibility, and robustness indicators

Finally, complementary insights about observational evidence can be obtained by examining how transparent and reproducible data and results are and how much trust one can place that analyses are robust to different modeling assumptions. It has now become evident that in many observational data sets, there are so many different ways to analyze the same data on the same question that the resulting spread of results (“vibration of effects”) [17] can allow almost any conclusion to be generated. Therefore, trust increases when the data are publicly available so that other analysts can examine them, when other analysts (including those with contrarian viewpoints) have analyzed them and reached the same conclusions, when the main inferences are not substantially modified with different modeling and analytical assumptions, and when it has been prespecified how analyses will be performed in a protocol that is ideally preregistered.

Table 3 shows these indicators for Khan and colleagues’ investigation and for two classic studies of air pollution and mortality, the Harvard Six Cities study [18] and the American Cancer Society study [19]. These classic studies made their detailed data available for reanalysis by an independent team that also included contrarian stakeholders in the design of the reanalyses, and reanalyses reached mostly similar conclusions [20], although there are still some dissenters. Conversely, the indicators of transparency and reproducibility are weaker in the mental health study.

**Table 3:** Transparency, reproducibility, and robustness indicators.

*Indicators*	*Mental Health*	*Mortality*
	*Khan and colleagues [4]*	*HSC [18], ACS [19]*
Data available to others		
Entirely unrestricted	No	No
Under specific conditions	Yes (*)	Yes
Reanalysis performed by others	No	Yes
Including contrarians	No	Yes
Results robust in different models	Mostly yes	Mostly yes
Prespecified protocol	No	Yes, not in full detail
Prespecified protocol for reanalysis	No	Yes

*Denmark data are available after approval only to investigators in Denmark. US data are available with payment; it is unclear whether the same exact data set as used by Khan and colleagues can be retrieved because new data continue accruing in IBM MarketScan.

**Abbreviations:**ACS, American Cancer Society study, HSC, Harvard Six Cities study.

Khan and colleagues should nevertheless be commended for addressing a number of suggestions that were raised during the peer-review process. The peer reviewers challenged the authors to show that their results remain largely similar with different analytical and modeling approaches. Therefore, several additional analyses were included during the course of the revision: internal validation in split samples, comparison of Poison regression versus Cox models, consideration of spatial correlation and autocorrelation in county-level data, and efforts to harmonize variables between the US and Denmark data. All these analyses give fairly consistent results to the original ones, thus enhancing the sense of robustness. However, one can envision additional analyses that could be pursued; e.g., it is not clear why only 4 psychiatric diagnoses are reported by Khan and colleagues when, in theory, dozens of psychiatric diagnoses (and thousands of medical diagnoses in general) can be analyzed in these rich data sets for association with air pollution. Furthermore, it is useful to have analyses also done by other investigators, including researchers who may have skeptical views about the association of air pollution and mental health.

These analyses, as well as subsequent studies in this field would benefit from rigorous, carefully prespecified protocols that are registered before the data are analyzed. Khan and colleagues have offered a brilliant exploratory analysis with interesting hypothesis-generating hints for bipolar disorder and possibly other psychiatric diagnoses. Now, these leads need to be rigorously prospectively evaluated in other data sets.

Finally, what does this quest for causation mean for public health and policy making? Certainly, air pollution has sufficient evidence to suggest that measures to contain it may save lives and decrease morbidity, e.g., from respiratory conditions, regardless of whether the causal strength of the association with mental diseases in particular is weak or substantial. However, if causal, this knowledge may also open new avenues to the prevention and treatment of mental conditions. Mental conditions carry a tremendous burden for individuals and society, and interventions to date have been only modestly effective.
